# Surgeons consider Rockwood classification the most important factor for decision‐making in acute, high‐grade acromioclavicular dislocations

**DOI:** 10.1002/jeo2.70203

**Published:** 2025-03-13

**Authors:** Eduard Van Eecke, Arno Alexander Macken, Derek van Deurzen, Thibault Lafosse, Antoon van Raebroeckx, Geert Alexander Buijze, Michel van den Bekerom, AC Instability Collaborator Group, Patrik Noll, Jorge Villa Sanches, Ken Lee Puah, Stefan Stanciugelu, Jeremy Munji, Etienne Lejeune, Kalman Piper, Claudio Rosso, Steven Corbett, Daniel Rojas, James Romanowski, Nyiko Chauke, Sophie Grosclaude, Youri Reiland, Michael Kimmeyer, Daniel Schwartz, Javiern Martin, Michael Thomas Freehill, Philippe Debeer, Saartje Defoort, Gaetan Opsomer, Ghislain Geurts, Stijn Hermans, Leon Diederix, Olivier Flamand, Antonio Harcha, François Melebeck, Alexander van Tongel, David Bassens, Florence Mulpas, Justine Barbier Michiel van Parys, Hans van der Bracht, Pieter‐Bastiaan De Keyzer, Dirk Petré, Wouter Jak, Rick Houben, Filip Robijns, Delphine Lambrecht, Luigi Banez, Lorenzo Castellani, Richard Jany, Vincent Wlodek, Alfonso Barnechea, Jennifer Mutch, Dhrumil Patel, Jose C. Minarro, Boukoros Evangelos, Christian Owesen, Ali Maqdes, C. P. J. Visser, Dominique Misselyn, Egbert J. D. Veen, Tim Kraal

**Affiliations:** ^1^ Department of Orthopaedic Surgery, Shoulder and Elbow Unit OLVG Amsterdam the Netherlands; ^2^ Department of Orthopaedic Surgery AZ Delta Roeselare Belgium; ^3^ Department of Orthopaedic Surgery and Sports Medicine Erasmus MC Rotterdam the Netherlands; ^4^ Clinique General Annecy, Alps Surgery Institute Annecy France; ^5^ Hand, Upper Limb, Peripheral Nerve, Brachial Plexus and Microsurgery Unit Clinique Générale Annecy Annecy France; ^6^ Department of Orthopaedic Surgery VITAZ Sint‐Niklaas Belgium; ^7^ Department of Orthopaedic Surgery, Amsterdam UMC University of Amsterdam Amsterdam the Netherlands; ^8^ Department of Human Movement Sciences, Faculty of Behavioral and Movement Sciences Vrije Universiteit Amsterdam Amsterdam the Netherlands

**Keywords:** acromioclavicular dislocation, shoulder, sports traumatology, survey

## Abstract

**Purpose:**

The aim of this study is to investigate the influence of patient‐specific factors, including age, lifestyle considerations as well as the extent of injury according to the Rockwood classification (RW), on the surgeon's decision‐making in the choice between operative and nonoperative treatment for acute, high‐grade acromioclavicular (AC) joint dislocations.

**Methods:**

Orthopaedic and trauma surgeons were requested to complete an online questionnaire consisting of closed and open questions regarding the treatment of acute, high‐grade AC joint dislocations and 24 fictive clinical scenarios.

**Results:**

A total of 133 answered questionnaires were collected. 27 different nationalities from five continents were represented. The included participants had a median experience of 12 years (interquartile range: 2–41). Overall, the treatment option for surgery (answer: YES) was chosen in 2426 answers (76% of cases) compared to ‘NO’ in 766 (24% of cases). RW classification was considered the most important factor influencing surgical decision‐making for most surgeons (69%). Two thirds of the participants answered that smoking does not impact their decision towards surgery and as to the influence of body mass index (BMI) on decision‐making, half of the respondents would not alter their preferred treatment based on BMI. Finally, there were no significant differences in decision‐making regarding the influence of the participant's demographics.

**Conclusion:**

This study highlights that RW classification is the most important factor to consider in the surgeon's decision‐making between operative and nonoperative treatment in acute, high‐grade AC joint dislocations. Participants preferred operative treatment over nonoperative treatment in acute, high‐grade AC joint dislocation in 76% of case scenarios, increasing up to 90% when RW Grade III lesions were not taken into account. These findings contrast with recent studies reporting good functional outcomes of conservatively treated acute, high‐grade AC injuries and highlight the need to bridge the gap between evidence and practice.

**Level of Evidence:**

Level V.

**Abbreviations**
AC: acromioclavicular
BMI: body mass index
IQR: interquartile range
RCT: Randomised Controlled Trial
RW: Rockwood

## INTRODUCTION

The acromioclavicular (AC) joint, a critical component of the shoulder complex, plays a pivotal role in maintaining the stability and functionality of the shoulder girdle [8]. Acute AC joint dislocations are common traumatic injuries, often resulting from sports‐related incidents, falls, or (motor) vehicular accidents [16]. Despite the availability of many therapeutic options, the injury of the AC joint and the choice of adequate therapy is still a great challenge today. AC joint injuries are classified according to the six‐graded Rockwood (RW) classification. There is a broad consensus regarding less severe injuries. RW I–II joint injuries are generally treated conservatively, without any treatment, paracetamol, nonsteroidal anti‐inflammatory drugs and/or short‐term shoulder immobilisation [13, 20]. Traditionally, RW Types IV–VI AC injuries are managed with surgery, while the treatment of Type III dislocations is still a topic of debate [11, 19]. However, in recent years, data has been published supporting non‐operative treatment of high‐grade AC joint dislocations.

In 2015, the Canadian Orthopaedic Trauma Society performed a multicenter randomised controlled trial (RCT), revealing no clear evidence that operative treatment with hook plate improves short‐term clinical outcomes compared to nonoperative treatment [12]. RCT by Windhamre found no difference in clinical outcome at 2‐year follow‐up in patients with Types III and V AC joint dislocation treated with hook plate or physiotherapy [2]. These findings were also seen in the RCT by Murray et al., which reports no functional benefit from operative over nonoperative treatment at one year following Type III or IV AC dislocations [14]. A Cochrane review by Tamaoki et al. reveals low‐quality evidence that surgical treatment has no additional benefits in terms of function, return to former activities, and quality of life at one year compared with conservative treatment [18]. Finally, Chang et al. published their meta‐analysis revealing no clinical difference in functional outcome between operative and nonoperative management of acute, high‐grade (III–V) AC joint dislocations. Patients in the nonoperative cohort had a more rapid return to work but were associated with a poorer cosmetic outcome [3].

Besides the indication for operative treatment, the timing of surgery is also debated. Haugaard et al. evaluated the results after acute AC joint dislocations RW Types III and V were treated non‐surgically with the option of delayed surgical intervention [6]. Ninety‐one per cent of patients recovered without surgery and there were no differences in outcome scores between Types III and V at any time point. Domos et al. mention the only advantage to operative intervention in acute settings, consistently borne out in the literature, is an increased probability of anatomic reduction. However, there is no correlation between reduction and improvement in pain, strength or motion [5]. These findings lead to a growing disagreement regarding the conventional beliefs that acute high‐grade AC joint dislocations have to be treated surgically.

The current decision‐making process behind choosing surgical treatment in acute high‐grade AC joint dislocations involves a complex interplay of various factors, including patient‐specific considerations, injury characteristics and surgeon experience.

This expert questionnaire study aims to comprehensively assess and analyse which parameters influence the surgeon's choice for operative treatment in acute high‐grade AC joint dislocations. It will explore patient‐specific factors, including age, lifestyle considerations (e.g., sports and occupation) as well as extent of injury according to the RW classification. Additionally, the study will investigate the impact of surgeon‐related variables, including experience and training background. We hypothesise that surgeon‐related variables, patient‐specific factors and the extent of injury would influence the decision‐making process for the management of acute AC joint dislocations.

## METHODS

Orthopaedic and trauma surgeons from different countries were requested to complete an online questionnaire consisting of closed and open questions regarding the treatment of acute AC joint dislocations and clinical scenarios. Participation was anonymous, and all scenarios were fictive; therefore, this study was not considered clinical research, and no ethics committee approval was required.

### Questionnaire description

The questionnaire was designed to gather data on the following: (1) participant's characteristics, (2) participant's opinion on the influence of age, smoking, body mass index (BMI) and timing of presentation on surgical decision‐making, (3) participant's treatment of choice (operative vs. nonoperative) for 24 short, fictional scenarios with acute AC joint dislocations (Appendix S1).

Expert characteristics such as years of experience, average annual number of AC joint stabilisations performed, subspecialty within orthopaedic/trauma surgery, type of hospital (public/private/academic) and nationality were collected.

In order to investigate the influence of the patient‐specific factors on the choice for treatment in acute AC joint dislocations, participants were asked to answer what they considered to be the maximum age to consider surgery for an acute AC joint dislocation. Next, they were also asked if elevated BMI (no/yes, if >30/yes, if >35) or smoking (yes, for all patients/no) would change their decision towards surgical treatment. At last, participants were asked what the maximum time from injury to surgery can be for which they would still consider performing a primary fixation (primary treatment as an ‘acute’ injury).

Following these questions, 24 scenarios in healthy, American Society of Anesthesiologists one to two patients with an acute AC joint dislocation were presented (Table 1 and Appendix S1). A description of the four variable factors (age, sports, occupation and RW classification) was provided for each scenario as well. Subsequently, the participants were asked if they would suggest surgical treatment for each case (Table 2). Finally, participants were asked which of the four they considered the most important factor in decision‐making.

**Table 1:** Scenario questionnaire.

	Age (years)	Sports	Occupation	RW
Case 1	20	Tennis	Construction worker	III
Case 2	20	Tennis	Construction worker	IV
Case 3	20	Tennis	Construction worker	V
Case 4	20	Tennis	Accountant	III
Case 5	20	Tennis	Accountant	IV
Case 6	20	Tennis	Accountant	V
Case 7	20	Recreational runner	Construction worker	III
Case 8	20	Recreational runner	Construction worker	IV
Case 9	20	Recreational runner	Construction worker	V
Case 10	20	Recreational runner	Accountant	III
Case 11	20	Recreational runner	Accountant	IV
Case 12	20	Recreational runner	Accountant	V
Case 13	38	Tennis	Construction worker	III
Case 14	38	Tennis	Construction worker	IV
Case 15	38	Tennis	Construction worker	V
Case 16	38	Tennis	Accountant	III
Case 17	38	Tennis	Accountant	IV
Case 18	38	Tennis	Accountant	V
Case 19	38	Recreational runner	Construction worker	III
Case 20	38	Recreational runner	Construction worker	IV
Case 21	38	Recreational runner	Construction worker	V
Case 22	38	Recreational runner	Accountant	III
Case 23	38	Recreational runner	Accountant	IV
Case 24	38	Recreational runner	Accountant	V

Abbreviation: RW, Rockwood.

**Table 2:** Would you suggest surgical treatment.

–	Yes
–	No

### Scenario selection

Age was identified as a potential factor of influence for the decision between operative and non‐operative treatment. The patient's age is known to affect the surgeon's decision‐making [17]. The patient's age was set at either 20 or 38 years in the fictional scenarios in the questionnaire.

Two other potential factors of influence are lifestyle considerations, being sports activity and occupation [5, 9]. A differentiation was made between overhead sports (tennis) and sports that do not stress the shoulder or AC joint function (recreational running). For occupation, a distinction was made between a job with physical activity/manual labour (construction worker) and a sedentary occupation (accountant). Finally, the grade of dislocation according to the RW classification was identified as a potential factor of influence. Traditionally, RW Types IV and V AC injuries are managed with surgery, while the treatment of Type III dislocations is still a topic of debate [19]. Scenarios were created with all possible combinations of these four variables.

### Participant recruitment and selection

All participants who completed part 1 (participants information) of the questionnaire and reported being an orthopaedic or trauma surgeon were included. Medical students, residents and other medical professionals were excluded.

### Statistics

Descriptive statistics will be used to report demographic data. The correlation between the case characteristics (independent variables) and the surgeon's choice (dependent variable) will be assessed using a Fisher exact test and pooling all cases with the same variable. All the statistical analyses will be performed using R/SPSS software (R Foundation for Statistical Computing). A *p* value lower than 0.05 was considered statistically significant.

## RESULTS

### Participants

In our study, a total of 133 answered questionnaires were collected. Twenty‐seven different nationalities from five different continents were represented in the questionnaire. The included participants had a median experience of 12 years (interquartile range [IQR]: 2–41), with the majority reporting to practice in a public hospital (*n* = 63, 47%), followed by private hospitals (*n* = 45, 34%) and academic hospitals (*n* = 25, 19%). The majority of the participating surgeons work as single joint‐focused shoulder surgeons (*n* = 73, 55%). Furthermore, the majority of surgeons reported performing 5–10 AC stabilisations each year (*n* = 49, 37%), followed by the surgeons that perform 10–20 AC stabilisations each year (*n* = 43, 32%).

These numbers are similar to the Delphi consensus study of Rosso et al. in 2021 among European Shoulder Associates–European Society of Sports Traumatology, Knee Surgery and Arthroscopy members [15]. About 54% of surgeons treated between 10 and 50 AC joints, whereas the other 46% treated less than 10 AC joints per year. Table 3 provides a complete overview of the participants' demographics.

**Table 3:** Participant characteristics (*n* = 133).

Type of orthopaedic surgeon, *n* (%)	
General orthopaedic surgeon	12 (9)
Upper extremity surgeon	36 (27)
Shoulder surgeon	73 (55)
Trauma surgeon	12 (9)
Years of experience, median (IQR)	12 (2–41)
AC stabilisations performed annually, *n* (%)	
<5	26 (20)
5–10	49 (37)
10–20	43 (32)
20–30	11 (8)
30–50	4 (3)
Type of hospital, *n* (%)^a^	
Public hospital	63 (47)
Private hospital	45 (34)
Academic hospital	25 (19)

Abbreviations: AC, acromioclavicular; IQR, interquartile range.

a Multiple answers were possible.

### Factors of influence

Regardless of all other patient or injury‐dependent factors, the median age limit for participants to consider surgical treatment for an acute, high‐grade AC joint dislocation was 61 years (IQR: 35–80 years). The option with no age limit was selected by seven participants (5%). When asked if smoking would change the decision toward surgical treatment in an acute, high‐grade AC joint dislocation, two thirds of the participants answered that this factor does not influence their decision towards surgery (*n* = 89, 67%). A minority would change their decision in all smokers (*n* = 16, 12%), and 21% of the participants (*n* = 28) would only change it if the patient is older than 30 years. Half of the participants would not alter their preferred treatment (*n* = 69, 52%) based on the patient's BMI. 40% (*n* = 53) would opt for non‐operative treatment in patients with BMI > 35 and 8% (*n* = 11) would opt for non‐operative treatment in patients with BMI > 30. For the maximum time from injury to surgery to consider performing a primary fixation (primary treatment as an ‘acute’ injury), the mean result was 3 weeks (IQR: 1–8 weeks). RW classification was considered the most important factor in decision‐making for 69% of the participants, followed by job (14%) and sports activity (11%). Age was indicated as the most important factor in only 6% of participants.

### Fictive scenarios

Overall, the treatment option for surgery (answer: YES) was chosen in 2426 answers (76% of all cases) compared to ‘NO’ in 766 (24% of all cases).

Regardless of taking other factors into consideration, surgery was found to be the participants choice for different scenarios with respective rates of 46% in RW Grade III scenarios (Cases 1, 4, 7, 10, 13, 16, 19 and 22), 90% in RW Grade IV scenarios (Cases 2, 5, 8, 11, 14, 17, 20 and 23) and 90% in RW Grade V scenarios (Cases 3, 6, 9, 12, 15, 18, 21 and 25). Remarkably, looking at the individual responses of each participant, in 89% of the cases, the same answer is given for a Grade IV or V injury, regardless of the other factors.

Respondents opted for surgery in the younger population (20 years) with a respective rate of 80% (Cases 1–12) as compared to similar scenarios with an older population (38 years) with a rate for surgical treatment of 71% (Cases 13–24). This difference was not statistically significant (*p *= 0.97). The only two cases in the younger group where more than half of the participants chose non‐surgical treatment were the 20‐year accountants with a Grade 3 injury (resp. rates 54% tennis or 63% recreative runner). In the 12 scenarios with ‘tennis player’ 79% of participants considered surgical treatment, whereas in the scenarios with ‘recreational runner’ 72% of participants considered surgery (*p* = 0.99). Regarding the influence of occupation on decision‐making, participants opted more often for surgical treatment in construction workers (79%) compared to patients who were employed as account (72%) in the different scenarios, but this difference was not significant (*p* = 0.99).

### Influence of participant's demographics

Overall, respondents working in a public hospital preferred surgical treatment in 78% of fictive case scenarios (1180/1512) compared to 74% in private hospitals (799/1080) and 72% in academic settings (431/600) (*p* = 0.09). When the responses of surgeons with more than ten years of experience are compared with those with less than 10 years of experience, our results have shown no significant difference in both groups (*p* = 0.87). When looking at the subspecialty of the respondents, a preferred surgical treatment was found among general orthopaedic surgeons in 79% of case scenarios (227/288), 76% among upper limb surgeons (655/684), 74% among shoulder surgeons (1301/1752) and 78% among trauma surgeons (225/288). These differences are not statistically significant (*p* = 0.71). When looking into the influence of surgical volume, our results have shown that surgeons performing less than <5 AC stabilisations/year, as well as the group with 5–10 AC stabilisations/year, preferred surgical treatment in 77% of case scenarios (480/624 and 902/1176). Surgeons performing 10–20 AC stabilisations opted for surgical treatment in 75% of cases (775/1032); for the group of 20–30 AC stabilisations/year, this rate is 74% (195/264), and lastly, the high‐volume group of 30–50 AC stabilisations/year opted in 78% of cases scenarios for surgical treatment (75/96). These minor differences are not statistically significant (*p* = 0.68).

## DISCUSSION

The main finding of this case‐vignette study is that RW classification is the most important factor to consider in the surgeon's decision‐making between operative and nonoperative treatment in acute, high‐grade AC joint dislocations. Participants preferred operative treatment over nonoperative treatment in acute, high‐grade AC joint dislocation in 76% of case scenarios, increasing up to 90% when RW Grade III lesions were not taken into account.

According to our results, the median age limit for surgeons still considering surgery in acute AC joint dislocations is 61 years. In the current literature, there is no consensus on a maximum age nor evidence that age has an influence on the outcome of surgical treatment in acute, high‐grade AC joint dislocation.

Smoking does only seem to alter treatment decisions towards surgery in our study in one‐third of the participants. However, a recent study by Choi et al. compared the postoperative coracoclavicular distance difference that was significantly higher in the smoker group (3.1 ± 2.6 mm) compared to the group with non‐smokers (1.7 ± 2.4 mm) [4]. Despite the radiographic differences, the postoperative clinical outcome scores and active range of motion measurements were comparable between the groups. Their retrospective study concludes that smoking does have a detrimental impact on ligament healing after hook plate fixation for acute AC joint dislocations and emphasises the importance of smoking cessation to optimise reduction maintenance after AC joint injury.

According to the literature, 3 weeks is generally the cut‐off value to consider an acute AC injury, whereas after 3 weeks, it is considered chronic [15]. Our findings correspond with this theory as a mean answer of 3 weeks for the time from injury to surgery (with a primary stabilisation without graft) was found. Interestingly, results by Ladermann et al. revealed equivalent clinical scores in both early and delayed surgical interventions, concluding rapid surgical intervention for high‐grade AC joint dislocation may not be necessary, as most patients can still benefit from surgery at a later stage [10]. All the patients in this study were managed using the same surgical technique of combined CC reconstruction and stabilisation of the AC joint, except for the addition of a gracilis allograft for biologic CC reconstruction in delayed intervention.

As is historically acclaimed, RW Types IV and V injuries are generally treated surgically with a variety of techniques aiming at anatomical reduction and realignment of clavicle and acromion. Our findings confirm this trend, stating that the majority of surgeons (76%) treat acute, Grades III–V AC joint injuries with surgery. This percentage is even higher when the grade III lesions are not taken into account (90%). The recent research of Haugaard, Windhamre and Murray reporting good functional outcomes of conservatively treated Grades III–V injuries questions our current approach and conventional belief that all acute high‐grade AC joint dislocations have to be treated surgically [2, 3, 6].

In 2013, Balke et al. published a German survey of trauma and orthopaedic departments revealing less specialised orthopaedic surgeons seem to have a stronger preference for recommending surgery for RWIII dislocations (75%) compared to more specialised arthroscopic/shoulder surgeons (69%). However, the reason for this difference remained unclear in their study [1]. Interestingly, a British survey by Domos et al. in 2018 also found that consultants who had less than 10 years of practice had a tendency towards earlier operative intervention compared to those who had 10 or more years of experience [5]. This trend was not observed in our results; there was no significant difference between surgeons with more or less than 10 years of experience.

### Limitations

This case‐vignette study has some limitations. Participation in the survey was restricted to orthopaedic and trauma surgeons. Emergency doctors/general practitioners/physiotherapists were not involved in the survey as they were less involved in setting the indication of operative treatment. Another limitation is the general limitation of a survey, such as low level of evidence (expert opinion) and leading survey questions. The strength of our study was that the included experts had a median of twelve years of experience in shoulder surgery from 27 different countries, thus allowing us to obtain a more diverse opinion on the subject. Currently, there are no guidelines or recommendations on the optimal sample size for expert questionnaire studies, nor are (too) small or large sample sizes clearly defined [5].

A subdivision into Grade IIIA versus IIIB AC lesions was not made, referring to the recent study of Haugaard stating the International Society of Arthroscopy, Knee Surgery and Orthopaedic Sports Medicine subclassification into stable IIIA and unstable IIIB lesions might not be clinically relevant [7]. Differences/variations in clinical examination were not mentioned in the individual case scenarios.

Additionally, the inter and intra agreement when assessing the RW grade is not taken into account. The result might differ when assessing the X‐rays compared to being told the RW classification.

A last limitation is the fact that only four factors in the fictive scenarios were included, possibly excluding the importance of other influencing factors. Other factors such as comorbidities (e.g., diabetes), associated fractures, associated nerve or vessel injury, subjective factors (patient choice), clinical assessment (skin puckering/tenting) or further differentiation into different levels of sports participation were not taken into account.

## CONCLUSION

This study highlights that RW classification is the most important factor to consider in the surgeon's decision‐making between operative and nonoperative treatment in acute, high‐grade AC joint dislocations. Participants preferred operative treatment over nonoperative treatment in acute, high‐grade AC joint dislocation in 76% of case scenarios, increasing up to 90% when RW grade III lesions were not taken into account. These findings contrast with recent studies reporting good functional outcomes of conservatively treated acute, high‐grade AC injuries and highlight the need to bridge the gap between evidence and practice.

## AC INSTABILITY COLLABORATOR GROUP

Patrik Noll (nollpatrik@gmail.com); Jorge Villa Sanches (jorgevillasanchez@gmail.com); Ken Lee Puah (puah.ken.lee@singhealth.com.sg); Stefan Stanciugelu (stefan.stanciugelu@umft.ro); Jeremy Munji (jemunji@gmail.com); Etienne Lejeune (lejeuneetienne@yahoo.fr); Kalman Piper (kalman@kaliper.com.au); Claudio Rosso (c.rosso@arthro.ch); Steven Corbett (steven.corbett@fortiusclinic.com); Daniel Rojas (danirojasmd@gmail.com); James Romanowski (jamroman@yahoo.com); Nyiko Chauke (chauke01.orthopaedic@gmail.com); Sophie Grosclaude (sophgc@gmail.com); Youri Reiland (yreiland@gmail.com); Michael Kimmeyer (michael.kimmeyer@vincentius-ka.de); Daniel Schwartz (daniel.g.schwartz@gmail.com); Javiern Martin (drjaviermartin@me.com); Michael Thomas Freehill (freehill@stanford.edu); Philippe Debeer (philippe.debeer@uzleuven.be); Saartje Defoort (saartje.defoortAsjki.be); Gaetan Opsomer (gaetanopsomer@yahoo.fr); Ghislain Geurts (ghislaingeurts@gmail.com); Stijn Hermans (stijnhermans@yahoo.com); Leon Diederix (lwdiederix@gmail.com); Olivier Flamand (drflamand@chirortho.be); Antonio Harcha (antonio.harcha.g@gmail.com); François Melebeck (fmelebeck@skynet.be); Alexander Van Tongel (alexander.vantongel@uzgent.be); David Bassens (david.bassens@azalma.be); Florence Mulpas (florence.mulpas@skynet.be); Justine Barbier Michiel Van Parys (michielvanparys@gmail.com); Hans Van der Bracht (hansvanderbracht@gmail.com); Pieter‐Bastiaan De Keyzer (pdkeyzer@azmmsj.be); Dirk Petré (drdirkpetre@proximus.be); Wouter Jak (wouter.jak@azturnhout.be); Rick Houben (rick.houben@telenet.be); Filip Robijns (filiprobijns@hotmail.com); Delphine Lambrecht (lambrecht.delphine@gmail.com); Luigi Banez (luigibanez@gmail.com); Lorenzo Castellani (lorenzo.cast@gmail.com); Richard Jany (richard.jany@ehnv.ch); Vincent Wlodek (vincent.wlodek@gmx.at); Alfonso Barnechea (alfonsobarnechea@gmail.com); Jennifer Mutch (j.mutch.ortho@gmail.com); Dhrumil Patel (mail.dhrumil1988@gmail.com); Jose C. Minarro (josecarlosdiaz10@gmail.com); Boukoros Evangelos (tempest2525@gmail.com); Christian Owesen (cowesen@gmail.com); Ali Maqdes (ali.maqdes@gmail.com); C. P. J. Visser (cpjvisser@alrijne.nl); Dominique Misselyn (dominique.misselyn@uzleuven.be); Egbert J. D. Veen (ejdveen@gmail.com); Tim Kraal (timkraal@hotmail.com).

## AUTHOR CONTRIBUTIONS

All authors contributed to the conception and design of the study, acquisition of data, analysis and interpretation of data. The article was critically revised for important intellectual content. The manuscript has been read and approved by all authors and each author believes that the manuscript represents honest work.

## CONFLICT OF INTEREST STATEMENT

Michel van den Bekerom and Derek van Deurzen report grants for clinical and research fellowships supported by Smith & Nephew. Geert Alexander Buijze reports consultant fees from Stryker. Thibault Lafosse reports consultant fees from Stryker, Smith and Nephew, Depuy. The remaining authors declare no conflicts of interest.

## ETHICS STATEMENT

Participation was anonymous and all scenarios were fictive, therefore, this study was not considered clinical research, and no ethics committee approval was required.

## Supporting information

Supporting information.

## Data Availability

The data that support the findings of this study are available from the corresponding author upon reasonable request.
