# Supplementary material for: Surgeons consider Rockwood classification the most important factor for decision‐making in acute, high‐grade acromioclavicular dislocations
Source: J Exp Orthop. 2025 Mar 13;12(1):e70203. doi: 10.1002/jeo2.70203 (PMC11904811; doi:10.1002/jeo2.70203)
Supplement: Supplementary file 1 — Supporting information. [file JEO2-12-e70203-s001.docx]

**Appendix 1 – Expert questionnaire**

ACUTE AC JOINT INJURY - EXPERT QUESTIONNAIRE

**WHAT INFLUENCES YOUR OPINION TO PERFORM SURGERY IN ACUTE AC JOINT DISLOCATIONS?**

This expert questionnaire study aims to comprehensively assess and analyze the **parameters influencing the surgeon's choice for operative treatment in acute AC joint dislocations**.

This is part of a research project by the Amsterdam Shoulder and Elbow Center of Expertise (ASECE) and Annecy Shoulder Research Group.

The survey should take approximately **5-10 minutes.**

Level of Expertise **Level of Expertise: What applies to you ?**

- General (orthopedic) surgeon (2)
- Upper extremity surgeon (3)
- Shoulder surgeon (4)
- Trauma surgeon (1)
- Other (6)

**Years of practice in shoulder surgery: What applies to you?**

Slide to the appropriate amount of years

|  | 0 | 5 | 10 | 15 | 20 | 25 | 30 | 35 | 40 | 45 | 50 |
| --- | --- | --- | --- | --- | --- | --- | --- | --- | --- | --- | --- |

| Years of experience () | 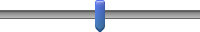 |
| --- | --- |

**Practice: What applies to you?**

I mainly practice in a (multiple answers possible):

- Public hospital (1)
- Private hospital (2)
- University hospital (3)

**Country: In which country do you practice?**

▼ Afghanistan (1) ... Zimbabwe (196)

**Amount of AC stabilisations performed each year: What applies to you?**

Each year I perform approximately:

- <5 AC stabilisations (1)
- 5-10 AC stabilisations (2)
- 10-20 AC stabilisations (3)
- 20-30 AC stabilisations (4)
- 30-50 AC stabilisations (5)
- >50 AC stabilisations (6)

**Regardless of all other factors, what do you consider the maximum age for which you would still consider surgical treatment for an acute, high-grade AC joint dislocation?**

Slide to the appropriate amount of years:

|  | No age limit |
| --- | --- |

|  | 0 | 10 | 20 | 30 | 40 | 50 | 60 | 70 | 80 | 90 | 100 |
| --- | --- | --- | --- | --- | --- | --- | --- | --- | --- | --- | --- |

| Age (years) () | 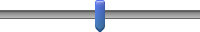 |
| --- | --- |

**Would smoking change your decision towards surgical treatment for an acute, high-grade AC joint dislocation?**

- Yes, for all patients (1)
- Yes, but only for older patients (>30y) (2)
- No (3)

**Would BMI change your decision towards surgical treatment for an acute, high-grade AC joint dislocation?**

- Yes, I would opt for non-operative treatment in patients with BMI > 30 (1)
- Yes, I would opt for non-operative treatment in patients with BMI > 35 (2)
- No, BMI does not change my preferred treatment (4)

**What is the maximum time from injury to surgery for which you would still consider performing a primary fixation (primary treatment as an ‘acute’ injury) ?**

|  | No limit |
| --- | --- |

|  | 0 | 1 | 2 | 3 | 4 | 5 | 6 | 7 | 8 | 9 | 10 | 11 | 12 |
| --- | --- | --- | --- | --- | --- | --- | --- | --- | --- | --- | --- | --- | --- |

| Time injury to surgery (weeks) () | 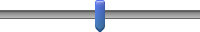 |
| --- | --- |

**Next you will be presented 24 short fictional cases and asked if you would perform surgery on this patient.  Please note that the cases may look similar but for each case one aspect is changed.**

**General information for all cases:**
- All patients present with an acute AC injury
- All patients are ASA 1-2, no significant comorbidities or previous shoulder injuries
- No associated fractures
- Upon clinical examination: lateral clavicle / AC tenderness, positive cross body adduction test

**20 years old, tennis player, accountant, Rockwood type 4**
Would you suggest surgical treatment? 

- Yes (1)
- No (2)

**38 years old, tennis player, accountant, Rockwood type 3**
Would you suggest surgical treatment? 

- Yes (1)
- No (2)

**20 years old, recreational runner, accountant, Rockwood type 5**
Would you suggest surgical treatment? 

- Yes (1)
- No (2)

**38 years old, recreational runner, accountant, Rockwood type 4**
Would you suggest surgical treatment? 

- Yes (1)
- No (2)

**20 years old, recreational runner, accountant, Rockwood type 4**
Would you suggest surgical treatment? 

- Yes (1)
- No (2)

**38 years old, tennis player, accountant, Rockwood type 5**
Would you suggest surgical treatment? 

- Yes (1)
- No (2)

**20 years old, recreational runner, accountant, Rockwood type 3**
Would you suggest surgical treatment? 

- Yes (1)
- No (2)

**20 years old, tennis player, construction worker, Rockwood type 3**
Would you suggest surgical treatment? 

- Yes (1)
- No (2)

**20 years old, recreational runner, construction worker, Rockwood type 3**
Would you suggest surgical treatment? 

- Yes (1)
- No (2)

**38 years old, recreational runner, construction worker, Rockwood type 4**
Would you suggest surgical treatment? 

- Yes (1)
- No (2)

**20 years old, recreational runner, construction worker, Rockwood type 4**
Would you suggest surgical treatment? 

- Yes (1)
- No (2)

**38 years old, recreational runner, construction worker, Rockwood type 5**
Would you suggest surgical treatment? 

- Yes (1)
- No (2)

**20 years old, tennis player, construction worker, Rockwood type 5**
Would you suggest surgical treatment? 

- Yes (1)
- No (2)

**38 years old, tennis player, construction worker, Rockwood type 3**
Would you suggest surgical treatment? 

- Yes (1)
- No (2)

**38 years old, tennis player, construction worker, Rockwood type 4**
Would you suggest surgical treatment? 

- Yes (1)
- No (2)

**20 years old, tennis player, construction worker, Rockwood type 4**
Would you suggest surgical treatment? 

- Yes (1)
- No (2)

**38 years old, tennis player, construction worker, Rockwood type 5**
Would you suggest surgical treatment? 

- Yes (1)
- No (2)

**38 years old, recreational runner, accountant, Rockwood type 3**
Would you suggest surgical treatment? 

- Yes (1)
- No (2)

**38 years old, recreational runner, construction worker, Rockwood type 3**
Would you suggest surgical treatment? 

- Yes (1)
- No (2)

**20 years old, recreational runner, construction worker, Rockwood type 5**
Would you suggest surgical treatment? 

- Yes (1)
- No (2)

**38 years old, recreational runner, accountant, Rockwood type 5**
Would you suggest surgical treatment? 

- Yes (1)
- No (2)

**20 years old, tennis player, accountant, Rockwood type 5**
Would you suggest surgical treatment? 

- Yes (1)
- No (2)

**38 years old, tennis player, accountant, Rockwood type 4**
Would you suggest surgical treatment? 

- Yes (1)
- No (2)

**20 years old, tennis player, accountant, Rockwood type 3**
Would you suggest surgical treatment? 

- Yes (1)
- No (2)

**What did you consider the most important factor in decision making?**

- Age (1)
- Sports (2)
- Job (3)
- Rockwood classification (4)

**Additional comments**

**__________________________________________________________________________**
